# Energy exchange: how we can personalize obesity therapy

**DOI:** 10.14341/probl12830

**Published:** 2021-10-14

**Authors:** O. V. Vasyukova, P. L. Okorokov, Yu. V. Kasyanova, O. B. Bezlepkina

**Affiliations:** Endocrinology Research Center; Endocrinology Research Center; Endocrinology Research Center; Endocrinology Research Center

**Keywords:** energy metabolism, basal metabolic rate, indirect calorimetry, double labeled water, obesity

## Abstract

Obesity is a consequence of chronic energy imbalance when energy intake constantly exceeds expenditure, which leads to excess white adipose tissue accumulation. Effective treatment of obesity requires accurate measure of calories intake and expenditure, as well as related behavior to understand how energy homeostasis is regulated and evaluate the effectiveness of the measures taken. The greatest interest is to study features of energy metabolism in various forms of obesity. It is necessary to create an evidence-based, personalized approach to diet therapy and to increase the effectiveness of weight loss measures. Modern studies have shown that the use of indirect calorimetry in obesity treatment programs leads to greater weight loss compared to traditional diet therapy planning based on calculated formulas.

## Abstract (ru)

Ожирение является следствием хронического энергетического дисбаланса, при котором потребление энергии постоянно превышает расходы, что приводит к накоплению избыточной энергии в виде белой жировой ткани. Для эффективного лечения ожирения нужны точные измерения потребления и расхода энергии, а также изучение связанного с этим поведения, чтобы понять, как регулируется энергетический гомеостаз и оценить эффективность проводимых мероприятий. Наибольший интерес вызывает изучение особенностей энергетического обмена при различных формах ожирения, что необходимо для создания доказательного, персонализированного подхода к диетотерапии и повышения эффективности мероприятий, направленных на снижение веса. Современные исследования показали, что использование непрямой калориметрии в программах лечения ожирения приводит к большему снижению веса по сравнению с традиционным планированием диетотерапии на основе расчетных формул.

Metabolism and energy exchange with the environment are a constant process for every human being. Human body regularly receives macro and micronutrients, water, and oxygen and regularly eliminates the end products of metabolism into the environment. Within cells, a continuous process takes place whereby organic substances are broken down and nutrients necessary for the formation and renewal of cellular components, tissues and organs are synthesised. In these processes, energy is released, adsorbed or stored in the form of macroergic compounds. Dynamic balance of metabolism is underpinned by the interrelationship of counterdirectional processes, i.e., anabolic ones (constructive metabolism) and catabolic ones. This interrelationship plays out as a complex, multi-layer regulation. Metabolism is heavily affected by the patient’s age, sex, racial group, health condition, nutrition status, daily physical activity, and the environment.

First studies in human energy metabolism were made in the 1780s as Antoine Lavoisier and Pierre de Laplace measured human oxygen intake and carbon dioxide output. They found that both values go up after eating and during physical exercises, though body temperature stays unchanged [1]. Lavoisier and de Laplace designed a small calorimeter for guinea pigs and showed a direct correlation between the amount of heat emitted by animals and their respiratory system. Those nascent experiments were further improved by Wilbur Atwater et al. who created a calorimeter suitable for investigations in humans and demonstrated that the most important law of thermodynamics – that of energy conservation – applies to human body. Thus, the amount of heat produced by a human body equals that emitted in the process of oxidising the food it intakes [2].

Thus, energy balance (the so-called zero energy balance) is achieved when the intake of energy useful for metabolism purposes matches the energy expenditure. Accordingly, non-zero energy balance means a unidirectional change of metabolism and body weight which then changes the energy expenditure. Thus, a positive energy balance causes the basal metabolic rate to increase as the body weight increases due to the increase of muscle mass required to maintain the spent depot fat, and vice versa: a negative energy balance reduces the body weight thus decreasing the basal metabolic rate. Assuming that the rate of energy spending stays unchanged after the initial intervention, one may expect that energy requirement will match energy consumption and the body weight will stabilise on a new given level [3].

However, those earlier axioms were challenged by research over recent decades. Obesity that has spread all over world is caused by chronic energy imbalance when energy intake continuously outweighs energy expenditure thus resulting in excess energy accumulation in the form of white adipose tissue. A seemingly simple solution is to recommend reducing the intake of calories (by abstaining from certain foods) and boosting energy expenditure (by increasing physical activity). However, contemporary studies show that these tactics are highly inefficient and suggest that obesity is much more complex a problem than it may seem at the first glance.

In addition, one has to admit that, on the one hand, recent fundamental studies using animal models and cell cultures have generated new knowledge in the domain of molecular pathogenesis which accounts for metabolic disorders and chronicity of the disease, but, on the other, they have not yielded any practical understanding as to the overweight management strategy, nor have they enabled medical science to develop efficient clinical measures to curb the obesity epidemic.

We are yet to fathom the sophisticated interrelationship between genetics, physiology and cognitive behaviour which jointly regulate the energy homeostasis.

At present, neither genetics nor biochemistry may be considered reliable predictors of body weight trend or disciplines facilitating accurate assessment of treatment efficiency. Given this, studies in energy metabolism as a sum total of various factors at work in an individual patient’s body have emerged as a rapidly growing area of obesity treatment personalisation.

Daily energy expenditure (DEE) in humans is comprised of three main components: basal metabolic rate, diet induced thermogenesis and energy expenditure for physical activity.

Basal Metabolic Rate or Resting Metabolic Rate (RMR) accounts for 55% to 70% of the body’s daily energy expenditure and represents the energy required to maintain the body’s essential functions, such as breathing, excretion, blood circulation, etc. [4]. RMR includes the body’s energy expenditure while at sleep and the energy required to keep the body functioning while awake without physical activity (the latter accounts for about 5% of RMR with slight variances depending on the patient’s racial group, sex, and weight). Lean body mass accounts for the better part of RMR (up to 70%).

Diet induced thermogenesis accounts for no more than 5% to 7% of the body’s daily energy expenditure and is the increase in daily energy expenditure above RMR caused by the body’s need to absorb and utilise the food it receives. Diet induced thermogenesis depends on the food component structure: diets high in protein are the most thermogenic, while those high in fat are the least so.

Energy expenditure for physical activity accounts for 25% to 30% of the body’s daily energy expenditure and represents the energy required to support both structured physical activity (such as sports and fitness) and daily physical activity.

Over the last 20 years, significant progress has been made in the assessment of human body’s energy expenditure; new methods and devices have been developed. Among the best known methods are direct calorimetry (heat emission measurements) and indirect calorimetry. The latter method is based on measuring the oxygen intake and carbon dioxide output, which also enables to measure the ratio of carbohydrates and lipids oxidised in the body.

The golden standard of total energy expenditure measurement is the method of double labelled water (DLW), first applied to humans in the 1980s [5–8]. The subject is administered water labelled with heavy but not radioactive isotopes of hydrogen (deuterium) and oxygen which then gradually leaves the body throughout the day (through kidneys, sweat, and exhaled air). This method is considered safe and is used in all age groups, including infants. Biofluid samples (blood, saliva or urine) are taken prior to and after the isotope administration and then once again at the end of the examination (one to three weeks after the start). The aforesaid hydrogen and oxygen isotopes content in the water obtained from such samples is then measured with a spectrometer. Since deuterium is eliminated from the body with water only whereas 18O is eliminated with both water and exhaled air, the rate of deuterium elimination is a measure of the body’s loss of fluid. The difference in the turnover rates of oxygen and hydrogen is a measure of the body’s energy expenditure. This method is quite labour-intensive and too complex to be used in routine clinical practice.

Findings of the largest DLW study conducted to date over a population were published in Science in August 2021 [9]. H. Pontzer at al. obtained unique data by analysing energy expenditure data in over 6,400 healthy male and female subjects residing in 29 different countries. The subjects’ age varied from 8 days to 95 years. It was found that metabolism level changes as human body grows, develops and ages. The basal metabolic rate was adjusted for fat-free mass so as to take into account the specifics of body composition, gender-specific properties and changes in metabolism status across the life span. As expected, total energy expenditure was found to increase with fat-free mass. The authors discovered four key periods in which substantial changes take place in human energy metabolism. The first period lasts from birth to 12 months of age. The infants’ metabolism (DEE and RMR) at first equals that of adults but then rapidly accelerates to almost double the adult level, reaching the peak at the age of 7 months. Following that, metabolism gradually slows down by the age of 20 years (no puberty-related metabolism acceleration was found in children aged 10 to 15) and stays at a plateau in adulthood (20 to 60 years) without any changes even in pregnancy and postpartum periods. With multivariate regression, male subjects aged 1 to 20 had higher metabolism levels than females. In older adult groups, sex was not found to have any substantial impact on the rate of DEE decrease with age. Finally, energy metabolism gradually declines in senior age even if the ratio of adipose tissue in the body remains unchanged. In addition, the frequency of chronic diseases was found to increase in that period (see Figure 1).

**Figure:**
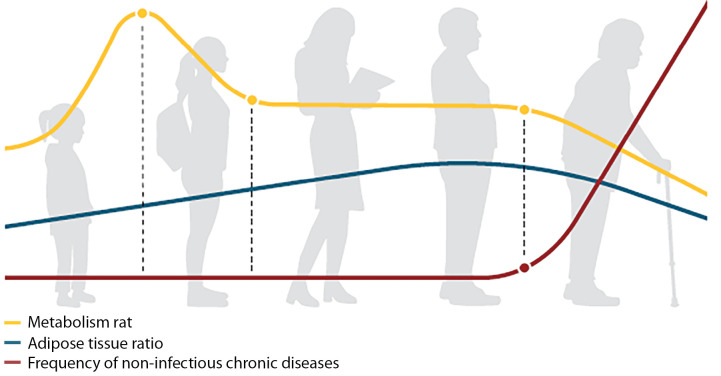
Figure 1. Metabolism across the life span [9]

In recent years, evidence has emerged that mitochondrial changes, nutrient supply and energy expenditure are interrelated, thus suggesting some kind of cell-level bioenergy adaptation. To overcome energy starvation, cell’s mitochondria increase the ratio of adenosine triphosphate (ATP) expressed per unit of nutrients received, whereas cells experiencing a surplus of nutrients increase energy waste in the form of heat emission through extrusion of protons from mitochondria [10]. In other words, human body adapts to changes in its energy balance by adjusting the efficiency of ATP synthesis. However, prolonged disorders (such as chronic positive energy balance with obesity) may cause mitochondrial dysfunction typical for metabolic diseases [11].

P. Balasubramanian et al. studied molecular aspects of the ageing process in animals; they concluded that reducing the diet’s caloric value helps curb various ageing-related processes in humans. First and foremost, this concerns several non-infectious diseases, such as cardiovascular disorders, tumours, sugar diabetes, dementia, osteoporosis, and sarcopenia [12].

Analysis of age-specific properties of metabolic status is important for personalising diet and physical activity recommendations, pharmacological correction and eventually, given H. Pontzer’s study which suggests that metabolic ageing probably does not begin until the age of 60, the search for predictors which may prolong the “metabolic youth” period.

The structure of human energy expenditure and the impact of age, environment, heredity background and lifestyle thereupon are still unclear. There are two hypotheses in this regard. The “accumulation” model presupposes that human body has some basic resting metabolic rate which increases with physical activity. The “limitation” model, on the contrary, postulates that the total expenditure level is fixed and any physical activity merely changes the ratio of energy expenditure associated with various needs of the body.

In 2019, H. Pontzer et al. studied the impact оf physical activity and infectious diseases on metabolism in children [13]. The authors assumed that the key properties of metabolism are shaped during the body’s rapid growth. They examined 44 Jivaroan children aged 5 to 12 (the entire Jivaro community numbers around 300). That indigenous community’s lifestyle is based on daily activities such as hunting and garden cultivation. These Indians have almost no access to electricity, water pipelines or medicine whatsoever. The control group was comprised of urban children from the USA and the UK. It was found that Jivaroan children spent almost double the time doing some physical activity compared to their urban counterparts. However, Jivaroan children’s ­energy expenditure for physical activity were almost two times lower. On the other hand, their RMR measured through indirect respiratory calorimetry was almost 20% higher. The authors believe it was due to a higher immunoglobulin G level and more formidable immune defence in the face of higher risk of infection. No substantial difference in lean mass was found between the two groups. Only a slight variance in total daily energy expenditure measured through double labelled water was found (p=0.258). Thus, the study confirmed the second energetic model, i.e., that of limitation. These data suggest that it is a change in the diet (the quality of energy supply) and not physical activity (energy expenditure) that makes the key contribution to energy imbalance which is the root of obesity prevalence. On the other hand, higher physical and immune activity in developing countries may deprive a child’s body of energy required for growth, even if the energy balance is positive.

The number one focus area is concerned with specifics of energy metabolism in patients having various forms of obesity. Such studies are needed to build an evidence-based personalised approach to diet therapy and to enhance the efficiency of weight reduction treatment.

Indirect respiratory calorimetry is recommended by Academy of Nutrition and Dietetics (USA) and American Academy of Paediatrics as a preferred method of resting metabolic rate measurement when planning diet therapy for children and adolescents with obesity [14][15]. S. Massarini et al. showed that the use of indirect calorimetry in obesity treatment programmes leads to more efficient weight reduction vs. the traditional diet therapy planning that uses formula-based resting metabolic rate calculation [16].

Various forms of obesity have their own specifics of energy metabolism. As regards the most widespread child obesity – exogenous – it ought to be said that normal RMR values are observed in 63.4%, low RMR in 25.8%, and high RMR in 10.8% of children [17]. Some authors believe that lower RMR is an adverse factor that contributes to obesity progress [18][19]. According to the most widely held theory, weight gains combined with insufficient increase of energy metabolism at rest and at the time of physical activity cause a positive energy balance even if no dietary disorders take place; this, in turn, leads to obesity progress. Such disorders may stem from hypothalamus and hormone dysregulation and from congenital or acquired defects of mitochondrial oxidation [20].

The literature offers contradicting views on the impact of changes in energy metabolism on obesity progress in children. G. Rodríguez et al. show that RMR adjusted for lean mass is lower in children with obesity compared to their peers with normal weight [21]. M. G. Hohenadel et al. provide data suggesting that RMR reduction in children under 10 is associated with subsequent obesity progress. However, prospective studies with observation time of 2 to 7 years have not confirmed any impact of RMR on chronicity of obesity in children [23][24]. Other studies have not found any correlation between RMR and subsequent weight gain in adults and children, whether having obesity or not having it [25–27], which points to the need of further prospective research to ascertain the impact of RMR value on subsequent obesity origin and course.

A widely held view was that RMR increase should be a favourable factor for obesity patients as it creates a negative energy balance. However, J. D. Cameron et al. demonstrated that in adolescents with obesity, RMR increase is associated with higher intake of calories and weight gain [28]. Thus, higher RMR cannot be deemed a favourable prognostic factor for obesity course in children and adolescents.

Morbid severe obesity is a special form of the disease which is particularly difficult to treat through lifestyle changes and diet therapy. A study in the specifics of energy metabolism in children showed that patients with morbid obesity have higher RMR values vs. the “non-morbid” obesity groups, regardless of the patients’ sex or lean body mass [29]. It ought to be noted that the use of calculation formulas leads to significant overstatement of RMR level in patients with manifest obesity, which may cause excessive food intake and low weigh loss efficiency; this is why the use of indirect calorimetry is preferred in such cases [17].

Hypothalamic obesity is a rare form of the disease. It occurs due to hypothalamic and brain axis tumours and their treatment, radiotherapy of brain tumours and haematological cancer, skull injuries or stroke. The main cause of hypothalamic obesity is a damage to the of parts hypothalamus which are involved in dietary behaviour regulation. Dysfunction of autonomic nervous system expressed as a reduction of sympathetic nervous system’s tonus is considered to be an important contributor to hypothalamic obesity origin, since sympathetic nervous system is directly involved in the regulation of the body’s energy metabolism [30]. Multiple studies have demonstrated that RMR reduction is one of the mechanisms which account for excessive weight gains in children with hypothalamic obesity [31–35]. I. Bomer et al. observed a significant RMR reduction (1,297 vs. 1,721 kcal; р<0.01) in a group of children who had craniopharyngioma surgically removed as compared to the control group of 43 children with exogenous constitutional obesity, where the control group’s sex, age and obesity degree parameters were commensurate with those of the target group [31]. Likewise, M. G. Shaikh et al. observed a RMR reduction in a group of 18 children with hypothalamic obesity [32]. The authors noted a reduction of locomotor activity in that group while no polyphagia was observed; they suggested that RMR and locomotor activity reduction are more significant factors causing weight gain in patients with hypothalamic obesity than the quantity of food they take. Average RMR reduction in patients with hypothalamic obesity has been found to be in the range between 13% and 17%; however, it may be as high as 33.4% in individual patients [32][33].

Syndrome forms of obesity in children occur due to chromosomal disorders, parental imprinting diseases or other genetic syndromes. The most frequent chromosomal pathology associated with obesity is Prader-Willi syndrome. The impact of energy metabolism disorders on obesity pathogeny in patients with Prader-Willi syndrome is widely discussed in the literature [36]. Most of the relevant studies find a significant RMR reduction (up to 37%) in patients with Prader-Willi syndrome vs. groups with simple (exogenous constitutional) obesity; at present, this difference is attributed to the lower lean body mass values in the former group [36–39]. Since Prader-Willi syndrome is also associated with hypopituitarism and hypogonadotropic hypogonadism, these factors also contribute to reduction of lean body mass and may intensify already existing energy metabolism disorders.

Thus, various forms of obesity are associated with their specific features of energy metabolism, and they are important factors to consider when developing personalised obesity treatment programmes.

## CONCLUSION

Human energy metabolism is a key factor affecting both the body’s daily dietary requirements and the body’s energy expenditure for various forms of activity. Analysis of the factors which determine the body’s energy expenditure is a highly complex task since the body’s composition, weigh, height and physical activity change through the life span.

Obesity is a disease caused by chronic energy imbalance. It has a heterogeneous structure: various forms of obesity differ by the impact of genetic, hormonal, metabolic or external factors. In addition, various forms of obesity are associated with their specific features of energy metabolism.

Efficient treatment of obesity requires accurate measurements of daily energy supply and expenditure and inquiries into the related behaviour to understand how energy homeostasis is regulated and to evaluate the efficiency of chosen tactics.

To date, several methods have been developed to measure various aspects of energy metabolism; each one has its advantages and disadvantages. Contemporary studies have shown that the use of indirect calorimetry in obesity treatment programmes leads to more efficient weight reduction vs. the traditional diet therapy planning that uses ­formula-based calculations.

## ADDITIONAL INFORMATION

Funding source. This study was funded by national mandate entitled “New Approaches to Personalised Treatment of Obesity in Children Based on Studies in Energy Metabolism, Beta Cells Reserve, and Expression of Adipokines, Myokines and Specific Chaperones”, reg. no. АААА-А20-120011790172-9.

Conflict of interest. The authors hereby declare no actual or potential conflict of interest related to this publication.

Authors’ contribution. Olga  V. Vasyukova, Yulia V. Kasyanova, and Pavel L. Okorokov: literature search and analysis; preparation of this article. Olga B. Bezlepkina: editing of this article and provision of substantial remarks. Every author approved the final version of the text prior to publication and agreed to accept responsibility for all aspects of this study, which implies due investigation and resolution of any issue related to the accuracy or integrity of any part thereof.
